# Structural Insights Support Targeting ASK1 Kinase for Therapeutic Interventions

**DOI:** 10.3390/ijms222413395

**Published:** 2021-12-13

**Authors:** Veronika Obsilova, Karolina Honzejkova, Tomas Obsil

**Affiliations:** 1 Department of Structural Biology of Signaling Proteins, Division BIOCEV, Institute of Physiology of the Czech Academy of Sciences, 25250 Vestec, Czech Republic; 2 Department of Physical and Macromolecular Chemistry, Faculty of Science, Charles University, 12843 Prague, Czech Republic; karolina.honzejkova@natur.cuni.cz

**Keywords:** ASK1, phosphorylation, MAP kinase, 14-3-3, kinase, protein–protein interaction

## Abstract

Apoptosis signal-regulating kinase (ASK) 1, a member of the mitogen-activated protein kinase kinase kinase (MAP3K) family, modulates diverse responses to oxidative and endoplasmic reticulum (ER) stress and calcium influx. As a crucial cellular stress sensor, ASK1 activates c-Jun N-terminal kinases (JNKs) and p38 MAPKs. Their excessive and sustained activation leads to cell death, inflammation and fibrosis in various tissues and is implicated in the development of many neurological disorders, such as Alzheimer’s, Parkinson’s and Huntington disease and amyotrophic lateral sclerosis, in addition to cardiovascular diseases, diabetes and cancer. However, currently available inhibitors of JNK and p38 kinases either lack efficacy or have undesirable side effects. Therefore, targeted inhibition of their upstream activator, ASK1, stands out as a promising therapeutic strategy for treating such severe pathological conditions. This review summarizes recent structural findings on ASK1 regulation and its role in various diseases, highlighting prospects for ASK1 inhibition in the treatment of these pathologies.

## 1. Introduction

Constantly exposed to a wide variety of stress signals, human cells sense stress through cellular sensors. These cellular stress sensors translate external information into appropriate cellular responses, such as survival, death, apoptosis and differentiation, according to the cellular context. Concomitantly, cellular stress sensors also contribute to whole-organism responses. However, any interruption of such stress responses can trigger imbalances underlying various diseases. Accordingly, stress signaling must be properly mediated through many biological intermediators, including kinases from the apoptosis signal-regulating kinase (ASK) family.

The ASK family consists of three family members, namely ASK1, ASK2 and ASK3. Since its discovery in 1997, ASK1 has been the most studied kinase of this family for its key role in the mechanism of stress- and cytokine-induced apoptosis [1]. This mitogen-activated protein kinase kinase kinase (MAP3K) is ubiquitously expressed. Unsurprisingly, diverse stress signals, namely oxidative and endoplasmic reticulum (ER), calcium influx and inflammatory signals, trigger its activity (reviewed in [2,3,4]). ASK2 and ASK3 are also involved in the regulation of important cellular processes, albeit with different outcomes [5,6,7]. ASK1-dependent cytokine production in inflammatory cells promotes tumorigenesis, whereas ASK2, in cooperation with ASK1, acts as a tumor suppressor [8]. Moreover, the ASK1/ASK2 heterocomplex regulates caspase-3 and poly (ADP-ribose) polymerase cleavage under stress conditions [9]. In turn, ASK3 participates in stress responses following several proapoptotic stimuli and regulates cell volume during osmotic stress [10,11]. In other words, ASK signaling determines cell fate depending on the interplay between its family members, particularly ASK1. 

**Figure 1:**
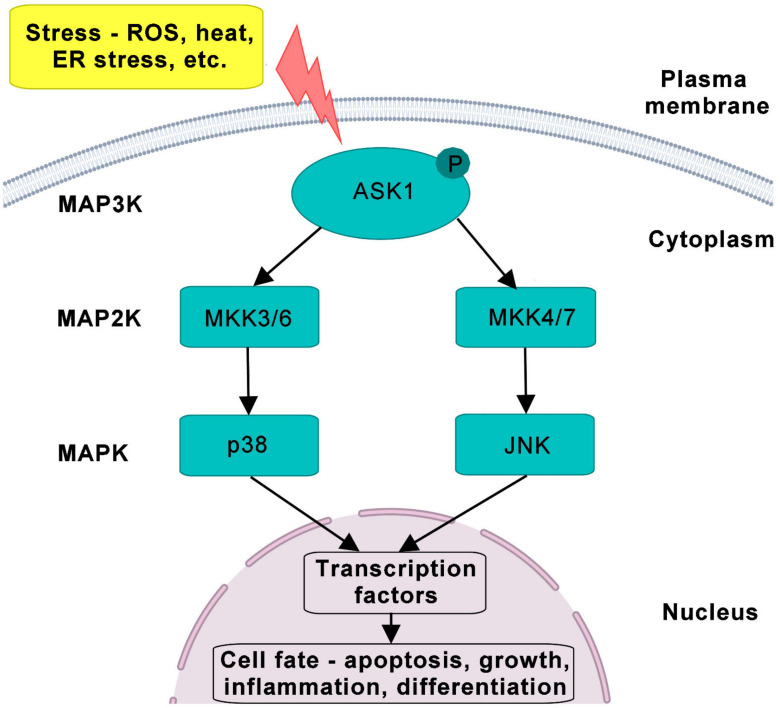
The MAPK cascade and its components. After various stresses such as ROS, ER stress, heat and bacterial infection, the activated form of ASK1 (MAP3K) activates MAP2K (MKK3/6 and MKK4/7) and consequently MAPK including p38 and JNK. All MAPKs are present in cytoplasm and can be shuttled to the nucleus to catalyze the phosphorylation of many proteins and transcription factors. The ASK1 pathway governs the cellular fate, such as apoptosis, growth, inflammation and differentiation. MAPK, mitogen-activated protein kinase; ROS, reactive oxygen species; ER, endoplasmic reticulum stress.

ASK1 signaling is regulated through a mitogen-activated protein kinase (MAPK) pathway involving three classes of protein kinases: upstream-activated MAP3Ks phosphorylate intermediate MAPK kinases (MAP2K), which subsequently phosphorylate terminal MAPKs (Figure 1). Activated MAPKs phosphorylate various kinases, receptors and transcription factors, thus regulating their activities and consequently their cellular responses. Three main groups of MAPKs have been identified so far: extracellular signal-regulated protein kinases (ERKs), c-Jun N-terminal kinases (JNKs) and p38 MAPKs [12]. ASK1 (also known as MAP3K5) belongs to the group of MAP3Ks that phosphorylate the MAP2Ks MKK4/7 and MKK3/6, which in turn phosphorylate and activate JNKs and p38 MAPKs, respectively [1]. Activated MAPK cascades then regulate cell proliferation, differentiation and cell survival. Furthermore, MAPK signaling is also regulated by various scaffolding and adaptor proteins (reviewed in [13]), which affect the subcellular localization and activity of protein kinases, thereby modulating downstream signaling.

Under non-stress conditions, the ASK1 homodimer and/or the ASK1/ASK2 heterodimer interact with dithiol oxidoreductases thioredoxin (Trx) and glutaredoxin (Grx) and the scaffolding proteins 14-3-3, forming a large multiprotein complex known as the ASK1 signalosome [14,15,16,17,18,19,20,21,22]. Both 14-3-3 and the oxidoreductases Trx and Grx function as negative regulators of ASK1 through mechanisms not yet fully understood [18,23,24,25,26,27,28,29,30]. Moreover, Trx binding induces ASK1 ubiquitination and degradation, further suppressing ASK1 activity [31]. Oxidative stress leads to the dissociation of Trx, Grx and 14-3-3 from the ASK1/ASK2 heterodimer, followed by binding to tumor necrosis factor receptor-associated factors (TRAF) 2, 5 and 6 [20,23,32,33]. The autophosphorylation of Thr^838^ within the activation loop then completes the process of ASK1 activation, which can then function as an upstream activator of JNK and p38 signaling.

Excessive and sustained activation of JNK and p38 kinases leads to cell death, inflammation and fibrosis in various tissues and is implicated in the development of many human diseases, such as cancer [34] and cardiovascular [35], inflammatory [36] and neurodegenerative diseases [37,38]. Because currently available JNK and p38 inhibitors either lack efficacy or have undesirable side effects [39,40], targeted inhibition of their upstream activator ASK1 remains the most promising therapeutic strategy to treat these severe pathologies, especially considering recent structural insights into ASK1 regulation.

## 2. Human ASK1 Structure and Regulation

**Figure 2:**
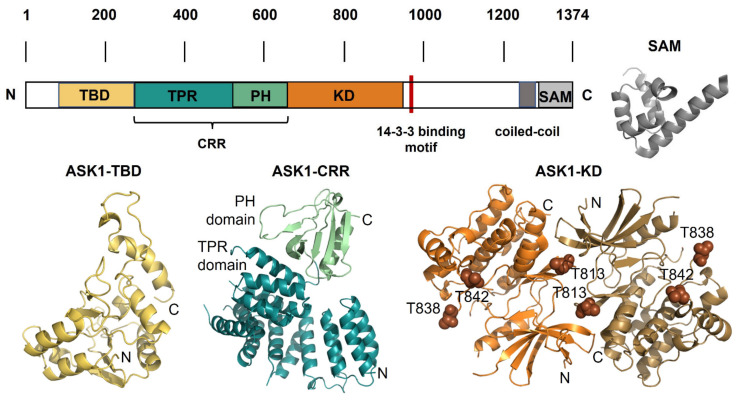
ASK1 and its structure. Schematic representation of the domain structure of ASK1 with the structural models of individual domains. TBD, thioredoxin-binding domain; TPR, tetratricopeptide repeat domain; PH, pleckstrin homology domain; CRR, central regulatory region; KD, kinase domain; SAM, sterile-alpha motif, the 14-3-3 site is shown in red. The structures of individual ASK1 domains: ASK1-TBD, the NMR-based structural model of thioredoxin-binding domain shown in yellow [29]; ASK1-CRR, the crystal structure of central regulatory region shown in teal and green (PDB ID: 5ULM [41]); ASK1-KD, the crystal structure of a dimer of kinase domain shown in orange and brown, and three autophosphorylation sites Thr^813^, Thr^838^ and Thr^842^ are shown as spheres (PDB ID: 2CLQ [42]); SAM, the crystal structure of SAM domain from ASK3 shown in grey (PDB ID: 6V0M [43]).

Human ASK1 is a 1374 amino-acid-long protein composed of several domains. The thioredoxin-binding domain (ASK1-TBD) is located at the N-terminus, followed by the central regulatory region (ASK1-CRR) and the kinase domain (ASK1-KD). In addition, the C-terminus of ASK1 also contains a 14-3-3 binding motif, a coiled-coil (CC) region and a recently identified sterile-alpha motif domain (ASK1-SAM) located at the C-terminus (Figure 2) [18,32,33,41,42,43]. Both ASK2 and ASK3 share a high sequence similarity to ASK1, especially within their kinase domains, with approximately 82 and 88% identity (amino acids), respectively [5,7].

### 2.1. ASK1-TBD

ASK1-TBD, located at the N-terminus of ASK1 between residues 46–277, is responsible for ASK1 binding to TRX, a physiological inhibitor of ASK1 and ASK1-dependent apoptosis [32]. Considering the biological functions of Trx, either reducing protein disulfide bonds or supplying reducing equivalents to redox enzymes, models have been proposed for ASK1 regulation based on a redox-dependent interaction with Trx [24,25,32]. Furthermore, the interaction between ASK1-TBD and Trx also induces ASK1 ubiquitination and its subsequent degradation, which is blocked by TRAF expression and tumor necrosis factor (TNF) treatment [31]. This tight control of ASK1-TBD highlights the importance of this domain for ASK1 function.

In solution, the isolated ASK1-TBD is a monomeric protein with a compact and slightly asymmetric shape, which binds Trx with a submicromolar binding affinity [26]. The recently reported NMR-based structural model of ASK1-TBD and its complex with Trx has suggested that ASK1-TBD adopts a fold resembling the thioredoxin structure, consisting of a central, six-stranded β-sheet surrounded by six α-helices and by an additional loop region (Figure 2) [29]. The Trx-binding surface of ASK1-TBD, formed by the ’seat’ and the ‘back-rest’ of the chair-like structure of ASK1-TBD, exhibits substantial conformational plasticity. However, the exact nature of this interaction between ASK1-TBD and Trx remains somewhat unclear. On the one hand, the intermolecular disulfide bond formed between ASK1 and Trx may promote a covalent interaction between ASK1-TBD and Trx [24,25,28]. On the other hand, weak non-covalent interactions may account for their reversible binding [26,29]. 

The binding affinity of Trx for ASK1 strongly depends on the redox state of Cys residues in its active site (Cys^32^ and Cys^35^ in human Trx1). Under reducing conditions, the residue Cys^32^ is responsible for the high-affinity association of Trx1 with ASK1-TBD [28]. Upon oxidation, disulfide bond formation between these two cysteines sharply decreases the binding affinity of Trx for ASK1 [20,28,31,32]. Because ASK1-TBD contains seven conserved cysteine residues, oxidative stress induces the formation of intramolecular disulfide bridges followed by a conformational rearrangement of the Trx-binding surface on ASK1-TBD [29], with Cys^250^ of ASK1 playing a key role in Trx binding [24,25,26,28,29,44]. This residue is located within the C-terminal region of ASK1-TBD, and its mutation to Ser considerably reduces Trx-binding affinity via structural changes throughout the domain, including the Trx-binding surface [28,29].

ASK1-TBD contains several sites of regulatory post-translational modifications. Protein kinase B (Akt)-, proto-oncogene serine/threonine-protein kinase PIM1- or p90 ribosomal S6 kinase 2 (RSK2)-mediated phosphorylation of Ser^83^ suppresses ASK1 activity by attenuating the binding of the ASK1 substrate MKK6 [45,46,47]. Conversely, phosphorylation of the second site Ser^174^, which is targeted by the Ste20-like kinase SLK, increases ASK1 activity [48]. ASK1-TBD also contains three methylation sites: Arg^78^, Arg^80^ and Arg^89^. The first two sites are methylated by protein arginine methyltransferase 1 (PRMT1), and their modification inhibits TRX dissociation and TRAF2 recruitment, thus reducing ASK1 activity [49]. The third site, Arg^89^, is methylated by PRMT5, and its modification promotes interactions between ASK1 and Akt kinase, thereby facilitating Ser^83^ phosphorylation and hence negatively regulating ASK1 [50]. 

### 2.2. ASK1-CRR

ASK1-CRR, the central regulatory region located between residues 269 and 658 of ASK1, binds to TRAF2, TRAF5 and TRAF6, adaptor proteins identified as ASK1 activators [19,33,51]. The structure of ASK1-CRR consists of two regions. The first region is comprised of 14 α-helices, which form seven tetratricopeptide repeat (TPR) motifs, whereas the second region consists of a pleckstrin homology (PH) domain (Figure 2) [41]. The TPR region of ASK1 differs from other TPR regions for its compact arrangement in which residues lie close to each other despite their separation in the primary sequence, in contrast to common extended arrangements. The PH domain is composed of two antiparallel β-sheets followed by a C-terminal amphipathic α-helix. It adopts a classical arrangement, except for the tryptophan residue commonly found within the terminal helix, which is absent in ASK1. Previously, an N-terminal coiled-coil motif responsible for mediating the oligomerization of the N-terminal region of ASK1 was predicted to be located between residues 297 and 324 [20]. However, the crystal structure of ASK1-CRR showed that this region was an integral part of the TPR domain, spanning the first and second TPRs [41]. Both the PH domain and the TPR region are required for ASK1 activity, not only by providing a binding site for TRAFs [20] but also by mediating substrate (MAPK2) binding and thus its phosphorylation through the adjacent ASK1-KD [41]. 

### 2.3. ASK1-KD

ASK1-KD adopts a typical protein kinase architecture formed by an N-terminal small lobe consisting of five β-sheets and an αC helix and a C-terminal, predominantly a large α-helical lobe [42]. The two lobes are linked by a hinge region, lining the catalytic ATP binding site. The domain is characterized by three autophosphorylation sites, Thr^813^, Thr^838^ and Thr^842^. These sites are important for ASK1 signaling, especially for Thr^838^ phosphorylation within the activation loop, which is an essential step to oxidative stress-mediated ASK1 activation. Thr^838^ is phosphorylated by murine protein serine/threonine kinase 38 (MPK38) or by transmolecular autophosphorylation [16,52]. 

The isolated ASK1-KD forms tight dimers in head-to-tail fashion, originating primarily from shape complementarity virtually across the length of the protein [42]. Whether this dimerization interface is also involved in the oligomerization of the full-length ASK1 remains unclear, but the kinase domain is positioned approximately at the center of the ASK1 molecule, C-terminally to the CRR domain. This kinase domain is followed by the 14-3-3 binding motif, whose Ser^966^ phosphorylation by PDK1 triggers binding to 14-3-3 proteins. 

These scaffolding proteins not only suppress ASK1 activity [15,18,21,30] but also stabilize the interaction between ASK1 and ASK2 [21,22]. Biophysical characterization of the complex between the ASK1-KD and 14-3-3ζ suggested that 14-3-3ζ interacts with ASK1-KD close to the active site. Consequently, this interaction may block accessibility to the active site and/or affect its conformation [27]. By contrast, 14-3-3 binding to ASK1 is inhibited by G(1) to S phase transition protein 1 (GSPT1), which interacts with ASK1 and enhances ASK1-induced apoptotic activity in a caspase-3-dependent manner [30]. 

ASK1-KD is also phosphorylated at Tyr^718^ by the kinase JAK2, thereby strengthening the association of ASK1 with the suppressor of cytokine signaling 1 (SOCS1), leading to ASK1 ubiquitination and degradation [53,54]. Moreover, ASK1-KD can also be modified by nitric oxide (NO) through the S-nitrosylation at Cys^869^. The effect of this modification depends on the conditions, that is, under cerebral ischemia-reperfusion conditions, NO activates ASK1; conversely, in IFN-γ-treated cells or under TLR signaling conditions, NO inhibits ASK1 by suppressing its interaction with MKK3/6 (reviewed in [3]).

### 2.4. ASK1-SAM

The latest insight into the structure of ASK1 was provided by Travelyan et al. [43], who identified a sterile-alpha motif (SAM) domain at the C-terminus of ASK1-3 proteins. SAM domains differs from others in their oligomerization propensity. In isolation, the ASK3-SAM domain forms stable oligomers, even at low protein concentrations; the ASK2-SAM domain is mostly found in its protomeric form; and the ASK1-SAM domain forms concentration-dependent oligomers. In mixtures, the ASK1- and ASK2-SAM domains promptly oligomerize into a stable heterocomplex. Therefore, these SAM domains presumably facilitate the formation of ASK1/ASK2 heterocomplexes. 

In line with the hypothesis that the mid-loop–end-helix (ML-EH) interface forms the oligomerization interface, mutations introduced into the ML-EH in both ASK3 oligomers and ASK1/ASK2 heterocomplexes prevent oligomerization. And while the crystal structure of ASK1-SAM has not been reported yet, its conformation is likely similar to the structure of ASK3-SAM, which adopts the classical five-helix fold of SAM domains (Figure 2) [43]. 

The C-terminal part of ASK1 also contains several other phosphorylation sites involved in ASK1 regulation. These phosphorylation sites provide additional levels of ASK1 regulation with potential physiological and pathophysiological implications in ASK1 signaling. For example, Ser^1033^ phosphorylation suppresses ASK1 activity [55]. In addition, Thr^1109^ and Thr^1326^ phosphorylation by RSK2 inhibits ATP binding to ASK1 [47]. 

## 3. Physiological and Pathophysiological Relevance of the ASK1 Signaling Pathway

ASK1 inhibition opens up a path towards curing many diseases. The role of ASK1 signaling in the development of various diseases and the therapeutic applications of the ASK1 inhibitors have been extensively analyzed in previous reviews [4,38,56,57,58,59]. Here, we present a brief overview of the role of ASK1 in cancer, diabetes and neurodegenerative, cardiovascular, kidney, liver, inflammatory and autoimmune diseases, in addition to discussing the prospects of ASK1 inhibition in treating these pathologies.

### 3.1. The Role of ASK1 in Neurodegenerative Diseases

Neurodegenerative diseases, such as Alzheimer’s, Parkinson’s or Huntington’s disease, are characterized by neuronal loss, accumulation of large quantities of misfolded proteins and toxic cellular effects. Their symptoms include visual and memory impairment and difficulties walking, talking and thinking, gradually progressing to the complete loss of these cognitive and motor skills. Oxidative stress is a common factor in the pathogenesis of these diseases. For this reason, therapeutic strategies for treating neurodegenerative disorders aim to reduce the activity of downstream signaling enzymes such as ASK1 [37,58].

#### 3.1.1. ASK1 in Alzheimer’s Disease (AD)

AD is the main source of dementia, primarily affecting people older than 60 years worldwide (reviewed in [60]). In AD, ASK1 is involved in amyloid-β-induced neurotoxicity and in endothelial and neuronal cell death, leading to cognitive impairment [61,62]. These neurotoxic processes result from the accumulation of abnormally folded amyloid-β, which induces neuronal death through ROS-mediated ASK1 activation [61]. Mutations in the amyloid precursor protein (APP) gene have also been associated with AD because they trigger the interaction between ASK1 and APP under cellular stress, as shown in 2-year-old 5XFAD ASK1-deficient mice (containing five AD-linked mutations) with improved cognitive function and decreased soluble amyloid-β in the brain [62]. Furthermore, the consumption of trans-fatty acids (TFAs), unsaturated fatty acids with at least one carbon–carbon double bond in a trans configuration, is also connected to neurodegenerative diseases, including AD, through a process involving ASK1. In their recent study, Hirata et al. [63] have demonstrated that extracellular ATP from injured cells induces the activation of the ASK1/p38 pathway and apoptosis downstream from the P2 × 7 receptor and ROS generation. Moreover, elaidic acid, the most abundant TFA from food, promotes ATP-induced apoptosis through abnormal activation of ASK1/p38 pathway via Ca^2+^/calmodulin-dependent kinase II (CaMKII) [63].

#### 3.1.2. ASK1 in Parkinson’s Disease (PD)

The key pathological feature of PD is the loss of dopaminergic neurons, which leads to motor disorders affecting up to 3% of the population older than 65 years (reviewed in [64]). In PD, ASK1 contributes to α-synuclein-induced neuronal damage and neuroinflammation, in addition to dopaminergic neuronal cell toxicity and death. Moreover, ASK1 is involved in 1-methyl-4-phenylpyridinium or 1-methyl-4-phenyl-1,2,3,6-tetrahydropyridine-induced cell loss in PD models. All these effects are associated with cognitive impairment [65]. Therefore, ASK1 is considered as a promising therapeutic target for preventing dopaminergic neuronal cell death.

The main genes related to PD are α-synuclein, leucine-rich repeat kinase 2 (LRRK2) and protein deglycase (DJ-1, also known as Parkinson disease protein 7). ASK1 is activated in α-synuclein transgenic mice in which ASK1 deletion reduces neuronal damage [66]. Recently, ASK1 inhibition also reduces apoptosis triggered by LRRK2, which is known to phosphorylate and activate ASK1 [67]. Mutations in another protein, DJ-1, are linked to recessively inherited PD, whereas DJ-1 WT has a protective role by sequestering the death domain-associated protein (Daxx) in the nucleus and thus prevents Daxx binding to ASK1 and its activation [68]. Corroborating these findings, Daxx trafficking inhibition with the SP600125 JNK inhibitor sufficiently decreases ASK1-mediated signaling in animal models, thereby highlighting the potential of this adaptor protein [69]. Furthermore, the receptor-interacting protein kinase 1 (RIPK1) is upregulated in patients with a neurodegenerative disease, and the recent study by Liu et al. [70] showed that the RIPK1 inhibitor Nec-1s has a neuroprotective effect against PD by inactivating the ASK1/JNK signaling pathway. 

#### 3.1.3. Huntington’s Disease (HD)

HD is characterized by progressive motor dysfunction, loss of cognitive function and psychical disturbance (reviewed in [71]). The average age at onset of HD is 40 years, with up to 20 years of survival. One of the modifiers of the age at onset of patients diagnosed with HD is the ASK1 gene [72]. The variability in this age at onset is also closely associated with ER stress, proteasomal dysfunction, polyglutamine (polyQ)-induced neuronal cell death and striatal cell loss induced by 3-nitropropionic acid.

HD is caused by an expansion of the CAG repeat in the huntingtin (htt) gene encoding a long stretch of polyQ, resulting in protein aggregation and ER stress, leading to ASK1 activation. Knocking down ASK1 in neurons suppresses JNK activation and cell death upon ER stress induction [73]. ASK1 inhibition also reduces ER stress and nuclear htt fragments in a mouse model of HD. Active ASK1 binds to htt fragments, releases them into the nucleus and consequently induces ER stress. Conversely, inactivated ASK1 binds to htt fragments, preventing their translocation to the nucleus and improving motor dysfunction and atrophy [74]. Concurrently, the JNK pathway also participates in HD pathology, as shown by the beneficial effects of blocking this pathway in a rat model of HD disease [75]. In addition, ASK1 is activated by a mitochondrial toxin, 3-nitropropionic acid, which induces oxidative stress and striatal damage similar to that found in HD [76]. Combined, these results support a direct link between ASK1 function an HD pathology.

#### 3.1.4. Amyotrophic Lateral Sclerosis (ALS)

ALS is a neurodegenerative disease with a fast progression to death (~4 years) after disease onset. Its neuropathology is characterized by the death of cortical and spinal neurons resulting in a progressive motor deficit. In ALS, ER stress is a key pathological feature of motor neuron death. Mutations in the copper–zinc superoxide dismutase (SOD1) are observed in 2% of all ALS cases, and familial ALS progression correlates with ER stress-induced ASK1 activation via a specific interaction of mutant SOD1 with Derlin-1, a component of ER-associated degradation machinery [77]. In canine degenerative myelopathy (CDM), which is considered a unique spontaneous large animal model of SOD1-mediated ALS in humans, the increased expression levels of ASK1 are associated with the upregulated expression of the ER stress marker GRP78/BiP in the spinal cord [78]. In a mouse model of ALS (SOD1^G93A^ transgenic mice), the selective inhibitors of ASK1 K811 and K812 developed by the Ichijo group significantly extended the life span of these animals [79], thus demonstrating that ASK1 inhibition holds a considerable therapeutic potential for the treatment of neurodegenerative diseases and for the prevention of cognitive disorders.

### 3.2. The Role of ASK1 in Other Diseases

#### 3.2.1. Cardiovascular Diseases

Cardiovascular diseases are some of the most prevalent causes of death worldwide. ASK1 plays a major role in cardiac hypertrophy and remodeling, mainly by promoting fibrosis. Meijles at al. [80] has recently shown that ASK1 inhibitors protect the heart from hypertension-induced cardiac remodeling, demonstrating the potential use of ASK1 inhibition in the treatment of hypertensive heart disease. Cardiac hypertrophy and remodeling are associated with angiotensin II signaling, increased intracellular Ca^2+^ and ROS production and the consequent activation of the ASK1/JNK/p38 pathways. Among the most important downstream targets of Ca^2+^ in hypertrophic signaling, CaMKII induces cardiomyocyte hypertrophy by activating the ASK1/NF-κB signaling pathway, whereas the CaMKII inhibitor KN93 inhibits phenylephrine-induced ASK1 activation [81]. Moreover, insulin-like growth factor-1 (IGF-1) directly affects cardiac cellular remodeling via the PI3K/Akt and ERK1/2 pathways. Three additional pathways known to be activated by IGF-1 have been recently identified: Rho-Associated Protein Kinase (ROCK), ASK1 and p38/MAPK. The activation of these pathways results in IGF-1-induced hypertrophy and collagen synthesis. Therefore, reducing ROCK, ASK1 and p38/MAPK activation could have cardioprotective benefits [82]. 

#### 3.2.2. Diabetes

Elevated blood glucose levels are the hallmark of the metabolic disease known as diabetes mellitus (DM), which affects over 400 million people worldwide. In DM, ASK1 is connected not only to pancreatic β-cell death, TNFα-induced insulin resistance and diabetic cardiac, retinal and renal dysfunction but also to loss of cognitive function [83]. In addition, JNK directly phosphorylates the insulin receptor substrate 1 under the activity of TNFα, increasing ROS production and ASK1 activation and leading to impaired insulin signaling. Considering these findings, ASK1 inhibition could be a therapeutic strategy for overcoming insulin resistance.

Diabetic patients with diabetic cardiomyopathy (DCM) show increased morbidity. Oxidative stress largely contributes to DCM progression, as shown in studies on mice in which the drug apocynin significantly suppressedASK1/p38/JNK signaling. According, this drug could be used as potential ASK1 inhibitor for treating DCM [84].

Neuropathy induced by DM contributes to a cognitive disorder termed diabetic encephalopathy (DE), which is also one of the risk factors for AD. ER stress is associated with the onset and progression of DE and AD. ASK1, JNK, TRAF2 and other factors associated with ER stress are upregulated in diabetic animals, but not in animals treated with Trx1, which could thus be a crucial factor for reducing DE by regulating ER stress and inhibiting apoptosis [85]. Moreover, upregulating Trx1 prevents diabetic hearing loss, most likely by suppressing ASK1 activation, thus further confirming the importance of ASK1 in DM-related pathologies [86].

#### 3.2.3. Liver Diseases

Liver diseases can be triggered by various apoptotic factors, such as hepatotoxins (chloroform, acetaminophen, thioacetamide), TNFα or the Fas ligand. ASK1 inhibition prevents liver inflammation, fibrosis and cell death. For example, ASK1 inhibition induced by glutathione-S-transferase overexpression reduced hepatocyte apoptosis induced by ASK1 overexpression or hepatotoxic agents such as thioacetamide and acetaminophen (APAP, also known as paracetamol) [87]. Several studies have recently reported that ROS-mediated liver injury induced by APAP causes severe liver collapse. APAP-induced damage in primary hepatocyte cultures results from ASK1/JNK activation, most likely through Trx dissociation from ASK1. Conversely, ASK1 deficiency protects against APAP-induced in vitro toxicity [88]. Furthermore, ASK1 inhibition using a combination of ASK1 inhibitors, N-acetylcysteine and GS-459679, protects against liver damage caused by APAP [89,90].

ASK1 inhibition has been shown to lead to liver protection under stress conditions, including a high-fat diet [91]. In addition, the progress of non-alcoholic fatty liver disease (NAFLD) depends on the regulation of the ASK1/p38/JNK signaling by TRAF1. Increased TRAF1 expression in the livers of NAFLD patients and TRAF1 overexpression in hepatocytes contributes to the development of insulin resistance, inflammatory response and hepatic steatosis. Conversely, TRAF1 deficiency is hepatoprotective [92]. Another liver disease termed non-alcoholic steatohepatitis (NASH) is also negatively affected by ASK1 signaling. This adverse effect can be reversed by a small peptide segment in CASP8 and FADD-like apoptosis regulator that inhibits the progression of steatohepatitis and metabolic disorders by disrupting ASK1 dimerization [93]. Moreover, the ASK1 inhibitors selonsertib and simtuzumab were recently tested in NASH clinical trials, which suggested that these compounds may reduce liver fibrosis in patients with NASH and fibrosis [94].

#### 3.2.4. Kidney Diseases

ASK1 is also a potential therapeutic target in renal fibrosis. Inflammation, hypoxia, mitochondrial dysfunction and other factors can worsen oxidative stress in an injured kidney. Reducing oxidative stress and inhibiting ASK1 may hence limit apoptosis, fibrosis and renal inflammation. 

A study of acute kidney injury in ASK1-deficient mice showed reduced levels of JNK activation and completely inhibited p38 activation [95]. ASK1 upregulation is also involved in other kidney diseases, for example, membranous nephropathy, which includes an immune reaction against glomerular epithelial cells. ASK1-mediated activation of p38 is a major factor of complement C5b-9 cytotoxicity in a membranous nephropathy model [96]. Moreover, treatment with the selective ASK1 inhibitor GS-444217 prevents proteinuria and glomerular thrombosis in glomerulonephritis [97]. Based on this evidence, ASK1 inhibition should be pursued as a therapeutic approach to protect kidney function by suppressing p38 and JNK activation to reduce tubular cell death, inflammation and fibrosis [98]. Moreover, diabetes is a major contributing factor to diabetic kidney disease (DKD) for which the ASK1 inhibitor selonsertib slowed diabetic kidney disease progression in a clinical trial with DKD patients [99]. Combined, these results demonstrate that targeted pharmacological inhibition of ASK1 protects renal function by decreasing cell death, inflammation and fibrosis.

#### 3.2.5. Cancer

The role of ASK1 as a tumor suppressor is a prevalent topic in the literature on ASK1 and cancer [34]. Variants of the ASK1 gene are identified with increased frequency in human cancer. Together, ASK2 and ASK1 function suppress tumorigenesis using the proapoptic activity in epithelial cells [8]. ASK1 alone also regulates the survival of both healthy and malignant plasma cells [100]. ASK1-regulating transcription factors, such as hepatocyte nuclear factor 4α, which binds to the ASK1 promotor and increases ASK1 expression, has a tumor suppressor function [101]. ASK1 is also involved in a tumor suppressor mechanism downstream of p53 that leads to the synthesis cytochrome c oxidase 2, which presumably dissociates Trx from ASK1 [102]. Yet another example is the pro-apoptotic functioning through the tumor suppressor DAB2IP, which inhibits the PI3K/Akt pathway and enhances ASK1 activation, ultimately promoting cell apoptosis in prostate cancer cells in vivo [103]. 

ASK1 also plays a pro-survival/pro-oncogenic role in tumorigenesis, for example, by phosphorylating and stabilizing the nuclear receptor TLX, thus inducing HIF-1α, a subunit of a heterodimeric transcription factor known as hypoxia-inducible factor 1 [104]. In this study, the authors also showed that siRNA knockdown of ASK1 expression down-regulated HIF-1α and the endothelial growth factor (VEGF-A) in neuroblastoma cells. ASK1 is also highly expressed in gastric cancer through increased cyclin D1 transcription, which contributes to tumor growth [105]. Another example of the pro-oncogenic role of ASK1 is the upregulation of the proinflammatory cytokine interleukin-6 (IL-6). In the resting state, ASK1 activity is inhibited by 14-3-3 proteins binding, among other factors. IL-6 mediates the dissociation of 14-3-3 from ASK1 (through Ser^966^ dephosphorylation), which leads to abnormal ASK1/p38 signaling and consequently to VEGF upregulation and angiogenesis elevation in human osteosarcoma [106]. IL-6 knockdown reduces VEGF expression and abolishes osteosarcoma-mediated angiogenesis. Lastly, ASK1 exhibits oncogenic activity in pancreatic cancer, where its expression correlates with the histological grade of pancreatic cancer, and its effect is abolished by ASK1 knockdown [107]. Therefore, considering the dual function of ASK1 in cancer, the oncogenic and anti-oncogenic roles of ASK1 must be understood in each cancer in order to target ASK1 in cancer treatments with inhibitors.

#### 3.2.6. Osteoarthritis (OA)

OA combines obesity and aging effects into the most common form of arthritic disease, affecting more than 250 million people worldwide [56]. The main features of OA are chronic pain and loss of joint function. Increased ROS production promotes chondrocyte proliferation, hypertrophy and apoptosis leading to increased articular cartilage. A recent study by Yan et al. [108] provided insights into the therapeutic potential of selonsertib, the inhibitor of ASK1, indicating that this drug could be used to treat OA. 

Particulate matter 2.5 (PM2.5) is an airborne particle originating from air pollution that enters the human body by diffusion into the blood. Very recently, Liu et al. [109] linked PM2.5 to the progression of OA through increased IL-6 production via the ROS, ASK1, ERK, p38, JNK and AP-1 signaling pathways. The authors suggested that ASK1 inhibition may be an effective strategy for reducing the production of proteins associated with cartilage catabolism and chondrocyte hypertrophy. Moreover, OA is characterized by chronic pain, and current findings show that OA pain stems from nociceptive inputs from damaged joints [110]. Stress-activated p38 and JNK are key contributors to chronic pain, with ASK1 contributing to both chronic and inflammatory pain. Because ASK1 KO mice show a protective phenotype against chronic pain, targeting p38 and JNK MAPKs with small molecule inhibitors could lead to effective therapeutic strategies for pain treatment (reviewed in [111,112]).

#### 3.2.7. Inflammatory and Autoimmune Diseases

One of the most common diseases among young adults is multiple sclerosis (MS), an inflammatory condition that affects the central nervous system. MS is an organ-specific, T-cell mediated autoimmune disease. In ASK1 knockout mice, the Harada group showed that ASK1 deficiency decreases neuroinflammation without affecting the proliferative capability of T cells in experimental autoimmune encephalomyelitis. Moreover, this study also found that several Toll-like receptors (TLR) were synergized with ASK1/p38 MAPK signaling in chemokine release. Therefore, targeting the TLR/ASK1/p38 pathway in glial cells is also a promising strategy for MS treatment [113]. 

Rheumatoid arthritis (RA), a common autoimmune disease that affects joints, is another example of ASK1 involvement in inflammation. ASK1 knockout mice are resistant to induced inflammatory arthritis in an RA model [114]. This study also showed that p38 and JNK inhibition block TNF-α induced production of IL-6 in synovial fibroblasts isolated from RA patients. In this context, ASK1 signaling pathways are induced by inflammatory cytokines through NF-κB activation [115]. 

## 4. Conclusions

In this review article, we summarized the current state of the art of ASK1 structure and the multiple roles of ASK1 signaling in various physiological and pathophysiological states, highlighting the prospects of its inhibition. Because excessive and sustained ASK1-mediated activation of JNK and p38 MAPKs in various stresses leads to the development of many human diseases, the precise knowledge of the signaling pathways involved, their regulatory mechanism and the physiological context at the molecular level are essential to the development of new treatment strategies. With a more comprehensive knowledge of the structure of ASK1, its interactions with binding partners within the signalosome (e.g., from cryo-EM analysis of full-length ASK1 complexes) and on mechanisms of ASK1 regulation, together with the development of specific small-molecule ASK1 inhibitors, we anticipate significant advances in pharmacological interventions targeting ASK1 in several diseases, ranging from cancer to neurological disorders.

## Abbreviations

AD	Alzheimer’s disease
Akt	Protein kinase B
ALS	Amyotrophic lateral sclerosis
APAP	Acetaminophen
APP	Amyloid precursor protein
ASK	Apoptosis signal-regulating kinase
CaMKII	Ca^2+^/calmodulin-dependent kinase II
CC	Coiled coil
CDM	Canine degenerative myelopathy
CRR	Central regulatory region
Daxx	Death domain-associated protein
DCM	Diabetic cardiomyopathy
DE	Diabetic encephalopathy
DJ-1	Protein deglycase
DKD	Diabetic kidney disease
DM	Diabetes mellitus
ER	Endoplasmic reticulum
ERK	Extracellular signal-regulated protein kinases
Grx	Glutaredoxin
HD	Huntington’s disease
HIF1	Hypoxia-inducible factor 1
htt	Huntingtin
IGF-1	Insulin-like growth factor-1
IL-6	Interleukin-6
JNK	c-Jun N-terminal kinases
KD	Kinase domain
LRRK2	Leucine-rich repeat kinase 2
MAP2K	Mitogen-activated protein kinase kinase
MAP3K	Mitogen-activated protein kinase kinase kinase
MAPK	Mitogen-activated protein kinase
ML-EH	Mid-loop–end-helix
MS	Multiple sclerosis
NAFLD	Non-alcoholic fatty liver disease
NASH	Non-alcoholic steatohepatitis
NO	Nitric oxide
OA	Osteoarthritis
PD	Parkinson’s disease
PH	Pleckstrin homology domain
PM2.5	Particulate matter 2.5
polyQ	Polyglutamine
PRMT	Protein arginine methyltransferase
RA	Rheumatoid arthritis
RIPK1	Receptor-interacting protein kinase 1
ROCK	Rho-associated protein kinase
ROS	Reactive oxygen species
RSK2	p90 ribosomal S6 kinase 2
SAM	Sterile-alpha motif domain
SOD1	Copper–zinc superoxide dismutase
TBD	Thioredoxin binding domain
TFA	Trans-fatty acids
TLR	Toll-like receptors
TNF	Tumor necrosis factor
TRAF	Tumor necrosis factor receptor-associated factor
Trx	Thioredoxin
VEGF-A	Endothelial growth factor
